# Subacute Cardiomyopathy Due to Statin Treatment: Can It Be True?—Case Report and Literature Review

**DOI:** 10.3390/life15040630

**Published:** 2025-04-09

**Authors:** Camelia Mihaela Georgescu, Ioana Butnariu, Cătălina Raluca Cojocea, Andreea Taisia Tiron, Daniela-Nicoleta Anghel, Iulia Ana-Maria Mitrică, Vlad-Iulian Lăptoiu, Adriana Bidea, Dana Antonescu-Ghelmez, Sorin Tuță, Florian Antonescu

**Affiliations:** 1 Faculty of Medicine, “Carol Davila” University of Medicine and Pharmacy, 050471 Bucharest, Romania; 2 National Institute of Neurology and Neurovascular Diseases, “Carol Davila” University of Medicine and Pharmacy, 041902 Bucharest, Romania; 3 Department of Neurology, National Institute of Neurology and Neurovascular Diseases, “Carol Davila” University of Medicine and Pharmacy, 041902 Bucharest, Romania; 4 Department of Cardiology, “Sf. Ioan” Emergency Clinical Hospital, “Carol Davila” University of Medicine and Pharmacy, 041902 Bucharest, Romania

**Keywords:** statin, myocarditis, cardiomyopathy, myositis, rhabdomyolysis, creatine kinase

## Abstract

Background and Clinical Significance: Statins are a widely used drug class associated with a plethora of muscular side effects ranging from the subclinical elevation of creatine kinase to fulminant rhabdomyolysis. Cardiac myopathy secondary to statin treatment is rare and was recently reported as a part of statin-induced necrotizing autoimmune myopathy (SINAM). Its occurrence outside of this context is still debated. Case Presentation: We present the case of a 60-year-old male who developed atorvastatin-induced rhabdomyolysis, without associated hydroxymethyl glutaryl coenzyme A reductase (HMGCR) antibodies, with clinical findings of cardiac failure and severe ECG anomalies. The symptoms slowly regressed with statin withdrawal, and the patient made a full recovery. We discuss the recently proposed statin-associated cardiomyopathy (SACM) and the possible mechanisms. We compare our case to the three other cases of statin-induced cardiac myositis found in the literature. Conclusions: We believe that in vulnerable patients, as was our case, statins can determine significant subacute cardiac toxicity. This would seem to occur in the context of severe skeletal muscle injury, probably due to higher metabolic resistance on the part of the myocardium. Also, the available evidence suggests myocardial involvement should be actively investigated in SINAM patients, preferably by cardiac MRI.

## 1. Introduction

Hydroxymethyl glutaryl coenzyme A reductase (HMG-CoA) inhibitors (statins) have been one of the most widely prescribed group of drugs in the world since their introduction to the market almost thirty years ago. They are used to treat hypercholesterolemia and prevent ischemic cardiovascular events, and although generally well tolerated, their administration can still be associated with many side effects, mostly minor, referred to as statin-associated symptoms (SASs) [1].

Statin-induced muscle injury lies on a spectrum, with most cases exhibiting only myalgias or slightly increased creatine kinase (CK) blood levels. In extreme cases, as in our report, they can induce severe rhabdomyolysis [2,3]. While statin-induced myopathy and rhabdomyolysis are well characterized, the myocardium is generally not affected, and the cause of this discrepancy is not readily apparent. We present a case of statin-induced acute cardiomyopathy associated with rhabdomyolysis and review a few other similar published cases.

## 2. Case Presentation

We report the case of a 60-year-old Caucasian male, a smoker, with chronic alcoholism and secondary Child–Pugh A liver cirrhosis, type 2 diabetes, hypertension, dyslipidemia, and peripheral artery disease type IIb Fontaine. Coronarography performed one year prior had shown single coronary vessel disease with a 99% stenosis of the middle segment of the right coronary artery.

The patient presented at our emergency department for lower limb weakness with difficulties walking and rising from a sitting position and mild myalgias in the lumbar area and thighs. The symptoms had appeared two weeks prior and had worsened progressively. Simultaneously with the neurological symptoms, the patient developed shortness of breath even with light effort. The patient denied any limitations of his daily activities before the symptoms appeared. A month earlier, the patient underwent a dental implant procedure. At that moment, he had ceased drinking and underwent a programmed cardiological re-evaluation, which recommended treatment with aspirin 100 mg/day, atorvastatin 80 mg/day, and propranolol 20 mg/day. Previously, the patient had not taken any medication for some years.

On admission, the neurological examination revealed predominantly proximal tetraparesis, more pronounced in the lower limbs; difficulty in raising the arms above shoulder level; and an inability to rise unaided from a sitting position. Walking was very difficult, requiring bilateral support. The vibratory sensation was reduced distally, with globally decreased deep tendon reflexes.

From a cardiological point of view, he had mild orthopnea but no other symptoms at rest. The patient denied chest pain or palpitations. No limb edema was present or reported. His heart sounds were rhythmic, with a 2/6 mitral systolic murmur. He was hemodynamically stable, with a BP of 139/73 mmHg, an HR of 74 bpm, and a peripheral oxygen saturation of 98%. No oxygen supplementation was required. The blood tests revealed mild thrombocytopenia and leukocytosis, elevated inflammation markers, significant muscle cytolysis, hepatic cytolysis, hyperammonemia, and hypocholesterolemia (Table 1).

**Figure 1:**
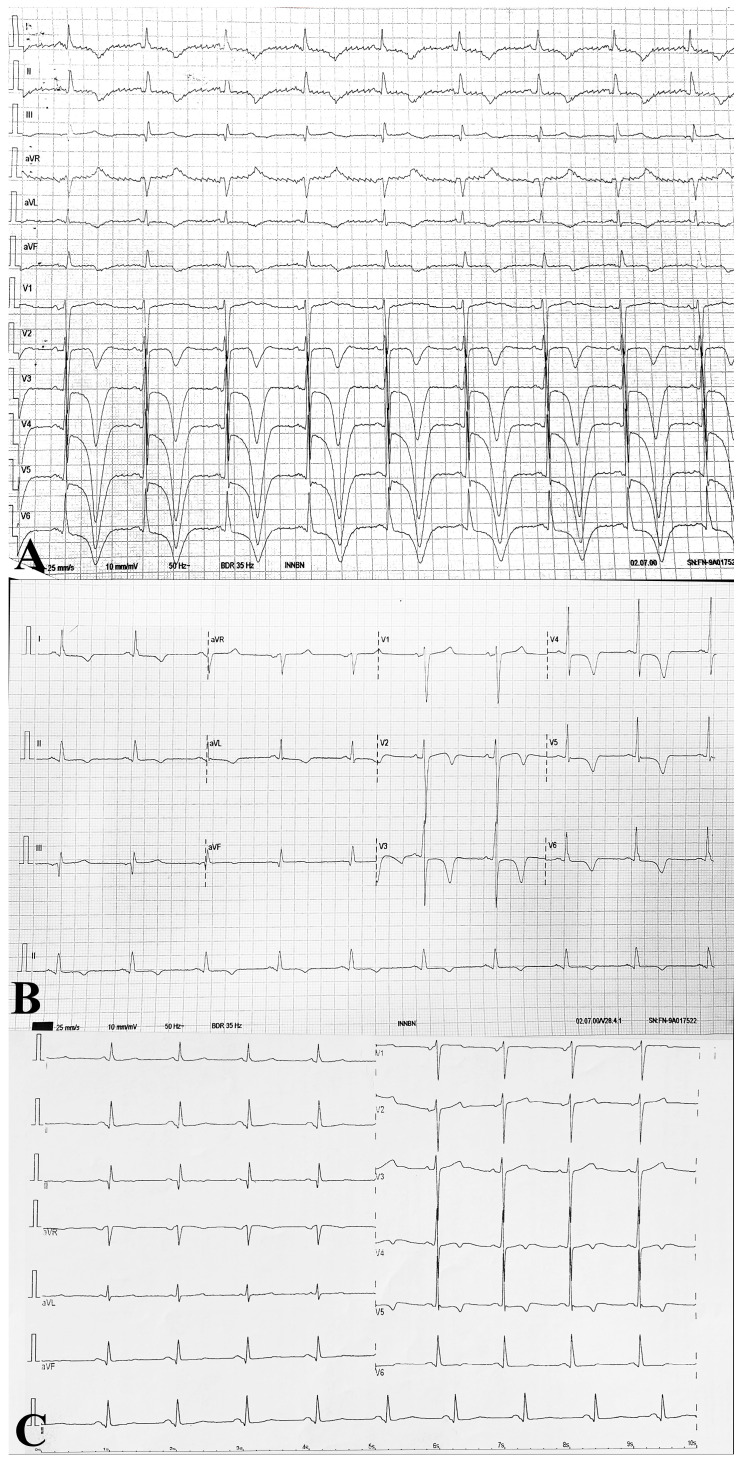
(**A**) Admission ECG shows sinus rhythm (HR 58 bpm), ST depression and deep negative T waves in V2–V6, generalized T wave inversion, q waves in the inferior leads, narrow QRS, no conduction disorders, left ventricular hypertrophy (Sokolow–Lyon index > 35 mm), and prolonged QTc (616 ms). (**B**) Day 7 ECG: sinus rhythm (HR 58 bpm), mild ST elevation (<0.5 mm) in V2, persistent but less pronounced negative T waves in V2–V6 and lateral leads, q waves in inferior leads, Sokolow–Lyon index of 37 mm, and QTc 500 ms. (**C**) Six-month follow-up ECG: sinus rhythm (HR 60 bpm), persistent q waves in inferior leads, and negative T waves in V4–V6.

An ECG showed a normal sinus rhythm without any conduction disorders, with ST-segment depression, negative T waves in leads V2–V6, and q waves in the inferior territory (Figure 1A). High-sensitivity troponin T and NT-proBNP values were elevated (Table 1). The troponin levels slowly decreased throughout hospitalization, without a dynamic suggestive of an acute coronary syndrome.

Transthoracic echocardiography showed concentric left ventricular hypertrophy with a ”salt-and-pepper” myocardial pattern, suggesting edema. Left ventricle function was impaired with diffuse wall motion abnormalities without specificity for a coronary artery territory. The ejection fraction calculated by the Simson biplane was 50%. The left atrium was of normal size. The right chambers had a normal systolic function. There was a grade II mitral regurgitation, type 1 diastolic dysfunction, and no pericardial effusion.

Since the patient denied chest pain, was hemodynamically stable, and the diffuse changes noticed on ECG and echocardiography were suggestive of cardiomyopathy rather than ischemia, a coronary angiogram was deferred, and he was monitored. No arrhythmias were noted during this period. We consider cardiac MRI an essential part of the diagnostic workup to objectively asses myocardial involvement. Unfortunately, this imaging modality is not available in our clinic. Referral to an external facility was considered, but the patient’s clinical status and the need for urgent treatment could not support the logistical constraints and anticipated delays in scheduling the examination.

Electromyography showed a diffuse myopathic pattern, with the spontaneous activity of fibrillations and positive sharp waves in the proximal muscles, especially in the lower limbs.

No history of familial hypercholesterolemia or hereditary myopathy was found. We considered viral myositis, but the patient had no fever or respiratory signs, and the blood lymphocyte count was normal. He tested negative for SARS-CoV-2 and HIV. Cytomegalovirus serology was negative for IgM and positive for IgG. Blood electrolyte levels were normal. A full panel for myositis was performed (Mi-2µ, Mi-2β, TIF1Y, MDA5, NXP2, SAE1, Ku, Pm/SCL 100, PM/Scl p75, Jo-1, SRP, PL-7, PL-12, EJ, OJ, and Ro-52) and was negative. Antinuclear and anti-HMGCR antibodies were negative. Another panel for common autoimmune diseases (lupus, scleroderma, and systemic sclerosis) was performed after discharge with no significant anomalies.

**Figure 2:**
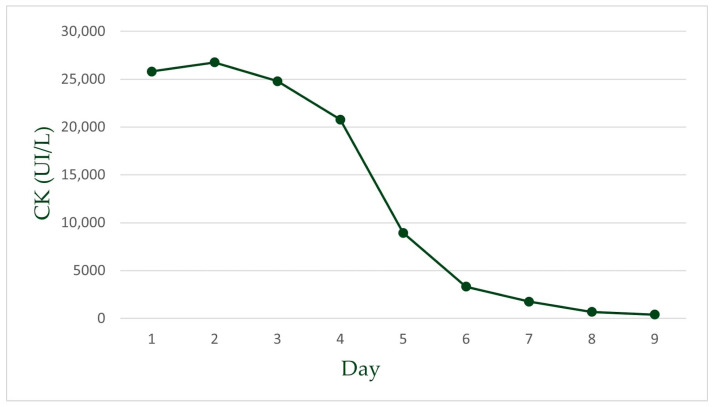
CK levels from day 1 to day 9 of admission. A progressive and substantial decline in CK levels was observed following the discontinuation of atorvastatin, consistent with recovery from muscle injury.

**Figure 3:**
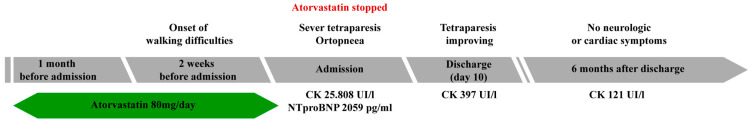
Timeline of symptom onset, atorvastatin treatment, clinical course, and biochemical markers. Atorvastatin at 80 mg/day was initiated one month prior to admission. The patient developed walking difficulties two weeks before admission, progressing to severe tetraparesis and orthopnea at presentation. Atorvastatin was discontinued upon admission. CK levels decreased from 25,808 U/L at admission to 397 U/L at discharge and had normalized by 6-month follow-up (121 U/L). NT-proBNP was elevated at admission (2059 pg/mL). Full neurologic and cardiac recovery was achieved in 6 months.

Atorvastatin was stopped, the patient was hydrated with IV saline solution, and treatment with methylprednisolone 1000 mg/day was administered for two days until rising glycemic values forced us to discontinue it. Muscle symptoms, ECG, CK, and hs-cTnT values progressively improved (Figure 1B, Figure 2 and Figure 3) during hospitalization. To complete the diagnostic workup, a myocardial biopsy was also considered. However, given the prompt and favorable response to treatment, it was decided that an invasive procedure with its associated periprocedural risk was not essential at that stage. Consequently, referral to another facility for biopsy was not pursued. We decided to discharge the patient after ten days with chronic treatment and follow-up.

At the six-month follow-up, the patient was in excellent condition, without any neurological or cardiac symptoms. The ECG showed a normal sinus rhythm at 65 bpm, normal PR and QTc intervals, minimal ST segment depression in V5, and inverted T waves in V4-V6 and D3, and q waves persisted in the inferior territory (Figure 1C). Echocardiography found a normal left ventricular function and reduced thickness in the wall muscle compared with the first examination, possibly reflecting remitted edema. Statins were not reintroduced. We recommended starting Ezetimibe, but he declined. PCSK9 inhibitors were not an option at that time due to financial limitations. The patient was referred for further coronarography, which he refused.

Initially, his personal experience was dominated by anxiety, especially regarding the near future and his ability to walk, but he tended to minimize the risks when they were explained to him and to brush aside the recommendations for future care. He was satisfied with the care he received, and his mood improved significantly when he was able to walk again. At the six-month follow-up appointment, his anxieties had waned, but he was still avoidant when the need for further coronarography was presented. When asked if he would ever consider restarting statins should he receive such a medical recommendation, he declined. The patient was monitored for a short period thereafter, but one year later, he discontinued follow-up due to complications from liver cirrhosis, which resulted in death.

## 3. Discussion

The main adverse effects of statins include muscle-related issues such as myopathy; liver effects (transient elevation of ALT is seen in about 2% of patients); and gastrointestinal issues like constipation, dyspepsia, and nausea. Since they are frequently encountered in practice, generally idiosyncratic, and sometimes dose-dependent, the preferred term is statin-associated symptoms [1]. However, serious adverse effects are generally rare, and the benefits of statin therapy in reducing cardiovascular events typically outweigh the risks. The major effect of statins is a reduction in blood LDLc, and it has been shown that each 1.0 mmol/L absolute reduction in low-density lipoprotein cholesterol is associated with a 20% reduction in the risk of cardiovascular events [4]. Research has also revealed statins have pleiotropic, anti-inflammatory, and antioxidant effects [5].

Statin-associated muscle symptoms (SAMSs) are the most common adverse effect of statin treatment, being reported by up to 25% of patients [1,6]. They are usually dose-dependent and, generally, tend to occur in the first three months of therapy [2]. The SAMS spectrum is considerable, extending from cramps and myalgias and/or slightly increased CK levels to subacute or chronic myopathy—which is reversible when the drug is stopped—and, in extreme cases, rhabdomyolysis [2,3]. It is estimated that only about 0.01% of patients develop an objective muscle injury with an increased CK level [7]. Multiple recent attempts have been made to define and classify SAMSs, out of which we find the SRM (statin-related myotoxicity) classification of the European Phenotype Standardization to be the most comprehensive [8].

Liver impairment is a known risk factor for statin side effects, including myopathy [9]. This derives from the fact that the main route of elimination for the majority of statins is through hepatic metabolization and bile excretion [10]. The fact that most statins (pravastatin excepted) are extensively bound to plasma proteins is especially relevant in patients with cirrhosis and hepatic insufficiency, where lower levels of plasmatic proteins increase the systemic exposure to the unbound, pharmacologically active fraction of the drug [11].

### 3.1. Statin-Associated Myopathy

The mechanisms underlying statin-induced myopathy are intricate, combining genetic predisposition, the multipronged effects of statins on cellular metabolism, the individual characteristics of each compound, and the patient’s immune system. The main triggering factor seems to be high statin serum concentration secondary to high-dose treatment or to associations with drugs that inhibit cytochrome P450 [12]. Statins with a lipophilic profile are known to have a higher risk of SAMSs, probably due to increased penetration through the cell and mitochondria wall [13]. Also, some transporter protein gene polymorphisms have been linked to an increased risk of statin-induced muscle injury [12].

HMG-CoA inhibition reduces mevalonate production and impacts the formation of other lipidic compounds, especially isoprenoids, which are involved in anchoring GTPases to the cell wall. This can impact cytoskeleton formation and internal signaling, leading to cellular dysfunction and possibly cell death [14,15]. This could explain the dose-dependent toxicity, as higher levels of HMG-CoA inhibition would affect the cellular membrane more severely. There are also possible lipid-independent statin effects leading to rhabdomyolysis. As such, 8-isoprostane levels, a known marker of oxidative stress, are increased in patients with statin-induced myopathy, with consequent normalization upon the discontinuation of treatment [15]. It is still uncertain whether this increase in oxidative stress is secondary to statin therapy and causal to the myopathy or is just secondary to the muscle injury.

Coenzyme Q10, also called ubiquinone, is an electron transporter in the mitochondrial electron transport chain, where it is involved in oxidative phosphorylation, a process required for the synthesis of adenosine triphosphate (ATP). It also has important roles outside the mitochondria, firstly as a lipid-soluble antioxidant, protecting cell membranes from oxidative injury, but also in the metabolism of pyrimidines and amino acids [16].

Statins reduce serum coenzyme Q10 levels, a phenomenon that seems to be both dose-dependent and amendable through oral supplementation [17]. It has also been reported that statins decrease CoQ10 levels in both cardiac and skeletal muscle [18]. This is probably a consequence of the fact that coenzyme Q10 is one of the products of the mevalonate pathway, which is inhibited at an early stage by statins blocking HMG-CoA reductase [19].

Coenzyme Q10 depletion decreases the phosphorylation potential of adenosine diphosphate and the activity of mitochondrial complexes I and IV, which contributes to mitochondrial dysfunction [20]. It also reduces the ability of cell membranes to resist oxidative stress [16].

Genetic factors seem to play an important role. Polymorphisms in the coenzyme Q2 gene, which plays an important role in the synthesis of coenzyme Q10, have been reported as predisposing patients to statin-induced myopathy [21]. From a functional perspective, it has been shown by Phillips et al. that statin therapy increases the respiratory exchange ratio, even in asymptomatic persons, and this may persist even after drug withdrawal [22,23,24].

CoQ10 supplementation ameliorates statin-associated muscle symptoms, implying that CoQ10 supplementation may be a complementary approach to managing statin-induced myopathy [20]. The effects of coenzyme Q10 supplementation for SAMSs are not clear, as available studies and meta-analyses have reported conflicting results [20,25,26]. The situation is further complicated by the fact that coenzyme Q10 has wide variations in bioavailability, depending on the formulation, and studies are heterogeneous regarding the chosen product [27]. There are also significant differences between individuals in the intestinal absorption of the drug [28].

### 3.2. Statin-Associated Cardiomyopathy

While the potential for skeletal muscle injury secondary to statin therapy is well recognized, cardiac muscle seems to be spared in the literature. It has been shown that the oxidative phosphorylation capacity of cardiac muscle exceeds that of skeletal muscle [29]. An excellent recent article by Somers et al. reviews the possible mechanisms behind this phenomenon [30].

Currently, the full effects of statins on the cardiac muscle and heart failure (HF) are under debate, but most of the literature supports a positive effect, especially in cases of HF with a preserved ejection fraction [31,32,33]. There is also available evidence that statins may help treat myocarditis and postmyocardial dilated cardiomyopathy, probably through an anti-inflammatory mechanism [34].

Recently, a new entity has been put forward. Statin-associated cardiomyopathy (SACM) is defined as an impairment in heart muscle function secondary to statin drug therapy of a severity sufficient to cause HF [31,35,36]. At the moment, SACM remains an extremely rare clinical entity, with just three cases recently described in the literature [3,6,37]. It is generally thought of as being a slow-developing, diastolic HF with preserved ejection fraction and is possibly responsive to CoQ10 supplementation, as the proposed mechanisms are statin-induced CoQ10 depletion with secondary impaired myocardial energy production and insidious cytotoxicity with sarcopenia [17,31,35].

It is conceivable that, as statins are frequently used in patients with ischemic cardiomyopathy and other forms of HF, detrimental effects on the cardiac muscle, especially if developing slowly, could be mistaken as a progression of the initial illness and thus considered only a “lack of efficiency”. It is possible that in cases of slowly worsening HF, without prior knowledge of a possible SACM, clinicians would be reluctant to try to stop statin treatment, as this may increase cardiovascular risk, and thus, the causality remains difficult to prove [31].

### 3.3. Statin-Induced Necrotizing Autoimmune Myopathy

Another novel entity has been identified and characterized in the last decade: statin-induced necrotizing autoimmune myopathy with anti-HMGCR antibodies (SINAM). It differs from other SAMSs by the fact that it tends to persist after stopping the offending drug and may require immunosuppressive therapy [38].

In cases of SINAM, it is assumed that in genetically susceptible patients, after an initial statin-induced autoimmune attack, the injured muscles release more HMGCR, perpetuating a cycle that becomes autonomous, thus explaining why it may not be abated by the discontinuation of statin and necessitates immunosuppressive therapy [3].

SINAM-associated cardiac myositis has been described. We are aware of three such cases reported in the literature, all recently, and all with associated myopathy (Table 2) [3,6,37]. In these cases, the probable mechanism was direct tissue injury not too different in its pathogenesis from the necrotizing myositis of the skeletal muscle [3]. All three patients were male and over the age of 70; all were under chronic statin treatment, which, in two cases, was mentioned as atorvastatin. One patient had a prior cardiac history of atrial fibrillation and diastolic HF. There was no mention of this topic regarding the other two patients. The type of onset varied, with two of the cases having a progressive development of symptoms over a few months, while the third had an acute onset. Echocardiography was normal in two cases, with septal hypokinesia and decreased left ventricle ejection fraction (LVEF) in the third. ECG was not presented for one patient, probably, we assume because it was unremarkable, and was without any significant modifications in the other two. Cardiac MRI was modified in all cases, the overarching feature being myocardial contrast enhancement.

### 3.4. Seronegative Statin-Induced Acute Cardiomyopathy—Our Report

To our knowledge, this is the first presented case of acute statin-induced cardiomyopathy with a negative HMGCR antibodies test. Unsurprisingly, it comes associated with an episode of rhabdomyolysis. Cardiac muscle is known to be more resistant to statin toxicity than skeletal muscle. Thus, reaching the threshold for cardiac damage likely implies severe skeletal muscle injury had already occurred [30].

Our argument for atorvastatin treatment as the common cause of the two is essentially temporal, as both muscle symptoms and cardiac anomalies developed simultaneously after treatment initiation and waned after its withdrawal.

The differential diagnosis of cardiomyopathy included endocrine disorders, autoimmune diseases, metabolic factors, coronary artery disease, infectious causes, alcohol intake, and hypertrophic cardiomyopathy. We excluded a metabolic, endocrinological cause or alcohol abuse due to normal laboratory markers and echocardiography findings, which showed no left ventricular dilatation. Hypertrophic cardiomyopathy was excluded due to the absence of asymmetric ventricular hypertrophy or mid-ventricular obstruction. Regarding previous coronary artery disease, although the patient had severe stenosis of the right coronary artery, the wall motion abnormalities were diffuse, without echocardiographic evidence of right ventricular or inferior wall impairment.

We ruled out an acute coronary syndrome due to the lack of angina, ECG, or echocardiogram changes compatible with coronary syndrome. The ECG showed nonspecific and non-territorial ST-segment and T-wave abnormalities, there were negative and deep T waves in all precordial leads and the inferior and lateral leads (compatible rather with dyselectrolytemia and myocyte impairment), and there was no pattern of abnormalities compatible with myocardial infarction with or without ST-segment elevation.

**Figure 4:**
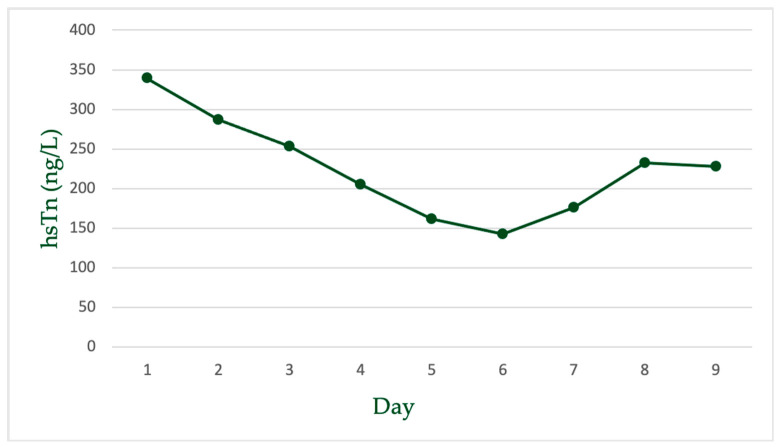
hs-Tn levels from day 1 to day 9. A gradual decrease in troponin levels was observed during hospitalization, followed by a mild rebound on days 7–9. This dynamic may reflect partial cardiac involvement with subsequent recovery after statin discontinuation.

High-sensitivity troponin T (hsTn) was elevated, but there was no rise-and-fall pattern, and the levels were less pronounced than what would be expected in a myocardial infarction (Figure 4) [39].

On echography, the left ventricle function was impaired with diffuse wall motion abnormalities without specificity for a coronary artery territory.

Also, cardiac tissue is generally spared in rhabdomyolysis, with rare exceptions as in secondary involvement as a consequence of severe metabolic disturbances or the particular toxicity of certain drugs, such as tyrosine kinase inhibitors [40]. Different cases presented in the literature illustrate different patterns of either initial myocardial injury with shock and subsequent rhabdomyolysis (especially after cardiac arrest or defibrillation) or severe rhabdomyolysis with cardiac involvement secondary to severe metabolic disturbances [41]. Acute kidney injury, electrolytic disturbances, and hemodynamic instability accompanying massive muscle cell lysis can all induce cardiac anomalies, but in our case, they were absent.

Given the severity of symptoms and the cardiac involvement, we decided against reinitiating statin therapy during the first year of follow-up. Still, even without rechallenging the patient with statins, we obtained a score of 8 on the Statin-Associated Muscle Symptoms Clinical Index (SAMS-CI) [42].

Diffuse ventricular wall motion abnormalities and increased ventricular wall thickness were initially present on the cardiac echography and remitted on follow-up. This contrasts with the above-presented cases of SINAM, which had normal echography in two out of three cases. The ECG was severely modified, again contrasting with the myocarditis with HMGCR antibodies.

### 3.5. Limitations

In our case, the diagnosis was primarily based on clinical presentation; ECG and echography findings; and, most importantly, on the onset following statin initiation and favorable evolution after statin discontinuation. Consequently, the diagnosis has several limitations. First, the patient had not undertaken any prior echocardiographic assessment, an investigation that could have determined whether left ventricular dysfunction was pre-existing or newly developed. Second, cardiac MRI was not included in our diagnostic workup due to a lack of availability at our clinic. MRI would have confirmed the myocardial involvement and helped to differentiate the SACM from other cardiomyopathies. Additionally, the absence of coronarography could have excluded an acute coronary syndrome. Finally, there was an absence of an endomyocardial biopsy, which could have also confirmed SACM.

## 4. Conclusions

The benefits of statin treatment are established [43]. The adverse effects of statins may be underrecognized by the medical community, especially since they are a commonly prescribed medicine, and doses are increasing as LDL-c targets are becoming more stringent according to new guidelines [4].

While the current literature supports the positive effects of statins on HF and myocarditis, our case represents a rare instance where they can act as offenders and result in potentially dangerous cardiac toxicity. All reported cases of acute cardiomyopathy have had prominent skeletal muscle involvement; thus, isolated cardiac toxicity is unlikely. Myocardial involvement should be actively investigated in SINAM patients, preferably by cardiac MRI, as transthoracic echocardiography and ECG seem to have a low sensitivity.

There also remains the possibility that the negative effects of statins on cardiac muscle are not necessarily rarer on the skeletal muscle but slower and more difficult to notice. This may also be confounded by the major benefits of statins, especially in patients with atherosclerotic cardiovascular disease.

Identifying vulnerable groups, such as the elderly and patients with cardiopathy, who are more susceptible to the adverse effects of statins, remains a significant challenge [30].

We think routine screening for SACM is advisable in patients with high-dose statin treatment, especially liposoluble drugs, who develop skeletal muscle symptoms. This screening should include ECG, echocardiography, and blood samples for evaluation of high-sensitivity troponin and NT-proBNP. Heightened clinical awareness and early cardiac imaging are essential in recognizing myocardial involvement early and preventing complications.

## Figures and Tables

**Table 1:** Significant blood tests at admission.

Tests	Results
Total CK	25.808 UI/l
CKMB	173 UI/L
hs-cTnT	339 ng/L
NT-proBNP	2059 pg/mL
C-reactive protein	23.8 mg/L
Total cholesterol	67 mg/dL
AST	1.061 UI/L
ALT	269 UI/L
PLT	100.000/µL
WBC	11.770/µL
Serum ammonia	82.8 µmol/L
Serum sodium	138 mmol/L
Total serum calcium	8.47 mg/dL
Serum potassium	4.2 mmol/L

Total CK—total creatine kinase; CKMB—creatine kinase-MB; hs-cTnT—high-sensitivity cardiac troponin T; NT-proBNP—N-terminal pro-b-type natriuretic peptide; AST—aspartate aminotransferase; ALT—alanine aminotransferase; PLT—platelet count; WBC—white blood cell count.

**Table 2:** Literature review of all cases of statin-induced acute cardiomyopathy and myositis (1–3). Our case (4) was added for comparison.

No.	Age/Sex	Onset	Myalgias	ECG	Prior Cardiac History	Anti HMGCR-Ab	hsTnT	Echocardiography	Cardiac MRI	Offending Drug
1 [3]	70 M	Months, sudden decompensation one week prior to presentation	Yes	Unspecified	Nonobstructive CAD; complete heart block with dual-chamber pacemaker	+	1619 ng/L	Normal	Myocardial edema Midwall late gadolinium enhancement	Chronic atorvastatin, 80 mg
2 [37]	71 M	Acute, 3 weeks prior to presentation	Yes	Not modified	No	+	211 ng/L	Septal hypokinesia LVEF 45%	Midwall late gadolinium enhancement of IV septum and LV wall	Chronic unspecified statin
3 [6]	80 M	Progressive, 3 months prior to presentation	No	Not modified	Hypertension, diastolic heart failure, and atrial fibrillation	+	900 ng/L	Normal LV systolic function with LVEF of 63%, no wall motion anomalies	Patchy mid-myocardial to epicardial Enhancement along the basilar septum and inferoseptal base	Chronic atorvastatin, unspecified dose
4 (Our report)	60 M	Subacute, 2 weeks prior to presentation	Yes	ST-T changes (Figure 1A)	PAD	-	339 ng/L	Diffuse wall motion anomalies LVEF 50%	-	Atorvastatin, 80 mg, started 2 weeks before onset

CAD—coronary artery disease; IV—interventricular; LV—left ventricle; LVEF—left ventricular ejection fraction; MRI—magnetic resonance imaging; PAD—peripheral artery disease, “+” denotes the presence of antibodies, “-“ denotes the absence of antibodies.

## Data Availability

The original contributions presented in the study are included in the article, further inquiries can be directed to the corresponding author.
